# Fused Deposition Modeling 3D Printing: Test Platforms for Evaluating Post-Fabrication Chemical Modifications and In-Vitro Biological Properties

**DOI:** 10.3390/pharmaceutics11060277

**Published:** 2019-06-13

**Authors:** Petra Arany, Eszter Róka, Laurent Mollet, Anthony W. Coleman, Florent Perret, Beomjoon Kim, Renátó Kovács, Adrienn Kazsoki, Romána Zelkó, Rudolf Gesztelyi, Zoltán Ujhelyi, Pálma Fehér, Judit Váradi, Ferenc Fenyvesi, Miklós Vecsernyés, Ildikó Bácskay

**Affiliations:** 1 Department of Pharmaceutical Technology, Faculty of Pharmacy, University of Debrecen, Nagyerdei körút 98, H-4032 Debrecen, Hungary; arany.petra@pharm.unideb.hu (P.A.); eszter.roka@gmail.com (E.R.); ujhelyi.zoltan@pharm.unideb.hu (Z.U.); feher.palma@pharm.unideb.hu (P.F.); varadi.judit@pharm.unideb.hu (J.V.); fenyvesi.ferenc@pharm.unideb.hu (F.F.); vecsernyes.miklos@pharm.unideb.hu (M.V.); 2 ICBMS, UMR 5246, Université Lyon 1, F69622 Villeurbanne, France; florent.perret@univ-lyon1.fr; 3 LMI CNRS UMR 5615, Université Lyon 1, 69622 Villeurbanne, France; laurent.mollet@univ-lyon1.fr (L.M.); anthony.coleman@univ-lyon1.fr (A.W.C.); 4 LIMMS/CNRS-IIS UMI 2820, Institute of Industrial Science, The University of Tokyo, Tokyo 153-8505, Japan; bjoonkim@iis.u-tokyo.ac.jp; 5 Department of Medical Microbiology, Faculty of Medicine and Faculty of Pharmacy, University of Debrecen, Nagyerdei körút 98, H-4032 Debrecen, Hungary; kovacs.renato@med.unideb.hu; 6 University Pharmacy Department of Pharmacy Administration, Faculty of Pharmacy, University of Semmelweis, Hőgyes Endre utca 7-9, H-1092 Budapest, Hungary; kazsoki.adrienn@pharma.semmelweis-univ.hu (A.K.); zelko.romana@pharma.semmelweis-univ.hu (R.Z.); 7 Department of Pharmacology and Pharmacotherapy, University of Debrecen, Nagyerdei körút 98, H-4032 Debrecen, Hungary; gesztelyi.rudolf@pharm.unideb.hu

**Keywords:** fused deposition modeling, polylactic acid, chemical modification, MTT assay, biofilm formation

## Abstract

3D printing is attracting considerable interest for its capacity to produce prototypes and small production runs rapidly. Fused deposit modeling (FDM) was used to produce polyvalent test plates for investigation of the physical, chemical, and in-vitro biological properties of printed materials. The polyvalent test plates (PVTPs) are poly-lactic acid cylinders, 14 mm in diameter and 3 mm in height. The polymer ester backbone was surface modified by a series of ramified and linear oligoamines to increase its hydrophilicity and introduce a positive charge. The chemical modification was verified by FT-IR spectroscopy, showing the introduction of amide and amine functions, and contact angle measurements confirmed increased hydrophilicity. Morphology studies (SEM, optical microscopy) indicated that the modification of PVTP possessed a planar morphology with small pits. Positron annihilation lifetime spectroscopy demonstrated that the polymeric free volume decreased on modification. An MTT-based prolonged cytotoxicity test using Caco-2 cells showed that the PVTPs are non-toxic at the cellular level. The presence of surface oligoamines on the PVTPs reduced biofilm formation by *Candida albicans* SC5314 significantly. The results demonstrate that 3D printed objects may be modified at their surface by a simple amidation reaction, resulting in a reduced propensity for biofilm colonization and cellular toxicity.

## 1. Introduction

Three-dimensional (3D) printing has become one of the major innovative technologies of recent years, and has led to a revolution in personalized medication using medical devices [1] and modified-release products [2]. The first solid dosage form (Spritam) of a medication printed by 3D technology was approved by the FDA in 2015. Nowadays, 3D printing methods may support the development of personalized medicine and therapy, for example in cardiac and orthopedic surgeries [3]. This innovative technique has also attracted significant attention in dental and plastic surgeries [4]. There are other technologies that are also being used for biomedical applications; for instance, fused deposition modeling (FDM) or stereolithography (SLA) [5]. Obviously, the first is used to prepare medical devices for surgical implantation, but is still onerous and highly expensive, whilst the second is cheap and suitable for prototyping devices both for biomedical analytical usage and clinical prototyping, such as drug patches [6]. For both FDM and SLA, commercial polymers or pre-polymers are now widely available, but little or nothing is known about their behavior in terms of bacterial colonization or biofilm formation, and indeed whether the printing process itself has an effect on the bio-properties of the post-printed structures remains to be clarified.

The concept of 3D printing in the medical field is aimed at designing anatomically correct prototypes using different imaging techniques, such as MRI, CT, and so forth, in which these pictures are transformed into file formats for 3D printers [7]. The possibility of producing individualized implants and oral drug formulations holds potential for a variety of different medical purposes. The use of 3D printing in the chemical sciences has expanded rapidly because different synthesis processes can be automatically controlled by, for instance, robot technics [8]. Glatzel et al. reported that a robotic setup may produce 3D printed antibacterial assays, which may promote new opportunities in the development of laboratory practice [9]. PLA-based implants are widely used due to their advantageous biodegradability and favorable cytocompatibility properties [10]. They also have good thermal plasticity and suitable mechanical properties [11]. Scaffold modifications/functionalizations have a great impact on the morphology and cytocompatibility of medical devices or 3D printed medicines. Side chain modifications may result in different surface properties [12]. Similarly, the use of surface absorption of polymers may allow modulation of the chemical and biological properties of 3D printed scaffolds, as in the very recent paper of Thire et al. on the use of polydopamine absorption onto 3D printed PLA scaffolds [13].

In a slightly earlier article, surface modification and the anti-microbial properties of the obtained systems were demonstrated. The previously utilized methods provided access to coated surfaces, however such systems can be expected to wear over time, which may limit their utility. Direct chemical modification of the polymers forming 3D printed objects would seem to be of interest, as the actual surface of the object is changed and not coated [14].

Of the available methods to characterize printed and modified PLA objects, FT-IR will definitively show the presence of new chemical functions, but intensities of the IR bands may be weak in the case of surface changes. Contact angle measurements will provide information on the hydrophilicity and hydrophobicity of the surface, and when correlated with FT-IR allow rapid characterization of surface chemical modifications. Scanning electron microscopy is an appropriate method for characterization of porosity and pore size of the surface of implants. Values of positron lifetimes and the corresponding intensities measured by positron annihilation lifetime spectroscopy may be connected with the size and the structural defects of implants [15].

To reach the general market with 3D printed implants, these devices must meet all standards and regulations defined by different authorities [16]. Biocompatibility investigation is a compulsory test of implantable devices. The ISO 10993-5 standard determines the parameters of the required cytotoxicity test. The main requirement is the time of contact duration: In the case of limited exposure the duration time is less than 24 h, and for prolonged exposure 1–30 days of investigation is required. Contact periods of longer than 30 days is regulated as requiring long-term studies [17]. Caco-2 cells are mainly used as a monolayer, rather than individual cells, however several assays are performed prior to reaching complete integrity, such as the end point or noninvasive cell viability assays (MTT assay, LDH test, real-time cell electronic sensing assay (RT-CES), etc.) [18]. MTT assay is a broadly used, rapid colorimetric method to measure the in-vitro cytotoxicity of certain compounds on cell lines or primary cells. It is usually performed in 96-well plates, thus is a high throughput method, which excludes cell counting. For decades it was considered as evident that MTT is transformed in the mitochondria, however in the last few years, doubts have arisen regarding the mitochondrial localization of the formazan formation [19].

Microbial biofilm formation could be a risk factor of implanted devices, potentially resulting in unpredictable complications [20]. These microbial biofilm formations may contribute to causing an inflammatory response and result in subsequent operations, such as dental operations. The ability of *Candida albicans* to form biofilms on medical devices could be a key property that enhances its ability to cause disease in humans [21]. Here, the use of chemical modification may allow inhibition of microbial biofilm formation, or may enhance such surface colonization. Interestingly, both growth inhibition and enhancement can be of considerable use to the researcher. A key point in such a modification is the presence of reactive ester groups in many of the polymers used in 3D printing, thus polylactic acid (PLA) and polyethylene terephthalate (PET) are based on polymers with ester backbones, whereas poly(methyl methacrylate) (PMMA) has ester side chain functions.

Many studies have been published on 3D printed implants and dosage forms, however, the connections between the chemical structures, structural parameters, and cytocompatibility results have not been well characterized yet. The objectives of our study were to find connections between the abovementioned parameters in case of chemically modified PLA based 3D printed polyvalent test plates platforms, or PVTPs. Various amines were used for the post fabrication chemistry to form active amide functionalities along the surface of 3D printed PVTP structures.

The structural parameters of the samples were evaluated using Fourier transform infrared (FTIR) spectroscopy, contact angle measurements, scanning electron microscopy (SEM), surface roughness measurement, and positron annihilation lifetime spectrometry (PALS). A cytotoxicity test based on an MTT assay on Caco-2 cells and investigation of biofilm formation with *Candida albicans* were performed to certify the cytocompatibility of the PVTPs. The work is a first step towards chemical functionalization of FDM and SLA 3D printed objects and also the study of the cytocompatibility of generic structures, and shows that simple structures can be produced rapidly and cheaply by 3D printing for use in biological experiments.

## 2. Materials and Methods

General: All chemicals were purchased from Sigma-Aldrich, Saint Quentin Fallavier, France and used without further purification. Deionized water was obtained using a Millipore (Merck Millipore Direct-Q 5 UV, Millipore SAS, Molsheim, France. 3D printer filaments were purchased from MakerShop Le Mans, France and stored under dry, dark conditions at room temperature.

### 2.1. Implant Manufacturing and Side Chain Modification

#### 2.1.1. 3D Printing Technique

The PVTPs were obtained using ColorFabb PLA Naturel filament of 2.85 mm [22]. PLA-PVTPs were printed using either a Lulzbot Taz 5 or a Lulzbot Mini 3D printer (Aleph Objects Inc., London UK). The print conditions are summarized in Table 1, below.

The PVTPs were designed using the Inventor program. The PVTP size was 14 mm diameter and 3 mm thickness. Batches of 25 were printed on the Lulzbot Mini and 50 on the Lulzbot Taz 3D printer. As with all FDM printing, initially adhesion happens to the print bed surface then to the previously printed layers, hence the importance of using the correct bed temperature for each source of filament [23].

#### 2.1.2. PLA PVTP Modification

The amidation reagents used were ethylene diamine (ED), triethylenetetramine (TET), *N*-methyl-1,3-propanediamine (NMePrN bis(aminopropyl)amine (BAPA), 2,2′ (ethylenedioxy)diethylamine (NPEGN), and tris(2-aminoethyl) amine (Tris). A total of 1000 mL 1 M solution of each amidation reagent was prepared in deionized water. No coupling reagents were added, as prior work using adenosine monophosphate had shown clean functionalization under the above conditions [24].

The PVTPs were placed in an in-house reactor, in this case a glass beaker, and 100 mL of the reactant solution was added. The use of beakers allows objects of dimensions up to 70 mm diameter and 50 mm height to be modified using low volatility reagents in closed aqueous conditions. The tops were closed and the system agitated on a rocking incubator for 24 h at 20 °C. The PVTPs were then isolated in a glass funnel and washed five times with 500 mL of deionized water. Finally, the implants were dried in air, under dust free conditions. The PVTPs were kept in air-tight containers prior to use [25].

### 2.2. Material Structure Characterisation

#### 2.2.1. FT Infra-Red Spectroscopy

Fourier transform infrared (FTIR) spectra of the PLA derivatives were recorded at room temperature by using an IRAffinity-1S FTIR spectrometer (SHIMADZU, Noisiel, France in the wave number range of 600–4000 cm^−1^. For these measurements, the samples were directly put on the attenuated total reflectance (ATR)-FTIR module (Miracle 10) and spectra recorder in the transmittance (%) mode [26].

#### 2.2.2. Contact Angle Measurement

Contact angle measurements were performed on a Kruss goniometer using DSA3 software (Lyon; France). A total of 10 µL of deionized water was deposited on the surface of the PVTP, and the droplet shape was imaged. The contact angle was determined using the internal Kruss software. Measurements were carried out a minimum of ten times for each PVTP, and the results were the average of the measurements [27].

#### 2.2.3. Scanning Electron Microscopy

SEM images were taken with a field emission-scanning electron microscope FE-SEM S-5000 (Hitachi Ltd., Tokio, Japan. Before SEM observation, 4 nm of palladium was deposited by atomic layer deposition on the treated surfaces, and they were put under vacuum overnight [28].

#### 2.2.4. Surface Roughness Measurement

The PVTPs were imaged using a VHX 6000 optical microscope (Keyence, Jonage, France, and the images were treated with the internal software to generate line profiles [29].

#### 2.2.5. Positron Annihilation Lifetime Spectrometry

For positron lifetime measurements, a positron source made of carrier-free ^22^NaCl was used, for which the activity was around 106 Bq. The active material was sealed between two very thin Kapton^®^ foils. The source was put between two pieces of implant. Lifetime spectra were measured with a fast–fast coincidence system based on BaF2/XP2020Q detectors and Ortec^®^ electronics. Three parallel spectra were measured from each sample. The spectra were evaluated by the RESOLUTION computer code. Three lifetime components were found in all samples, and the longest component was identified as positronium triplet state (o-Ps) lifetime [30].

### 2.3. Cytotoxicity Experiments

#### 2.3.1. Cytotoxicity PLA-Based PVTPs Sterilization

PLA-based PVTPs were immersed in 70% (*v/v*) ethanol in a laminar air flow (LAF) cabinet for 12 h, and were taken into sterile mull paper individually for drying [31].

#### 2.3.2. Cell Culture

The colon adenocarcinoma (Caco-2) cell line was received from the European Collection of Cell Cultures (ECACC, Salisbury, United Kingdom, catalogue No.86010202). Caco-2 cell line is a well-established cell culture in our laboratory. As it can be seen in the article of Nemes et al. the cell culture is used in based on the same protocol. We state that the Caco-2 cell line provenance is from ECACC. Cells were seeded in plastic cell culture flasks (Thermo Fisher Scientific Inc., Budapest, Hungary in DMEM medium supplemented with 3.7 g/L NaHCO_3_, 10% (*v/v*) heat-inactivated fetal bovine serum (FBS), 1% (*v/v*) non-essential amino acids solution, 1% (*v/v*) l-glutamine, 100 IU/mL penicillin, and 100 µg/mL streptomycin at 37 °C in an atmosphere of 5% CO_2_. For the cytotoxic experiment, cells between 20–40 in passage number were used. The culture media was replaced with fresh media every three or four days [32].

#### 2.3.3. MTT Cell Viability Test

Cells were seeded on flat bottom 96-well tissue culture plates at a density of 10^4^ or 3 × 10^4^ cells/well. After sterilization of the test samples separately, they were put in sterile centrifuge tubes and immersed in 2 mL of the DMEM medium. The samples were stored in a cell incubator at 37 °C. The test was performed on the 4th, 8th, and 12th day, while the samples were stored under the same conditions. The first step of the MTT assay was to remove the culture media from the cells, then the cells were treated with 200 µL of the test sample solution and incubated for 30 min. After the incubation time, the samples were removed and the cells were washed with 200 µL PBS solution/well. Then, the cells were incubated with 0.5 mg/mL MTT dye. Finally, the formazan crystals were dissolved in acidic isopropanol (isopropanol: 1.0 N hydrochloric acid = 25:1). The absorbance was measured at 570 nm against a 690 nm reference with a FLUOstar OPTIMA Microplate Reader (BMG LABTECH, Offenburg, Germany). Cell viability was expressed as the percentage of the untreated control [33].

#### 2.3.4. Biofilm Formation

In brief, aliquots of 500 µL of standardized SC5314 cell suspension (1 × 10^6^ cells/mL) in Roswell Park Memorial Institute (RPMI) 1640 medium (Sigma Laboratories Ltd., Budapest, Hungary) were added into flat bottom 12-well tissue culture plates containing implants (diameter of 1.6 cm), and incubated at 37 °C for 24 h [34]. Afterwards, the samples were taken into a new 12-well plate and incubated with 0.1% crystal violet solution for 10 min, and the bounded crystal violet was dissolved with 30% acetic acid. The dissolved solution was taken into a flat bottom 96-well tissue culture plate, and absorbance was measured at 540 nm in an Anthos HTII spectrophotometer (Anthos, Salzburg, Austria) [35].

### 2.4. Statistical Analysis

Data were analyzed using GraphPad Prism (version 7.0; GraphPad Software Inc., La Jolla, CA, USA) and presented as means ± standard deviations (S.D) of triplicates. Comparison of the groups was performed by a one-way ANOVA in STATISTICA software version 13. 2. (Statsoft, Tulsa, OKUSA) at a confidence level of 95%; results were regarded as significant at *p* < 0.05. All experiments were carried out in triplicate and repeated at least three times.

## 3. Results

### 3.1. Implant Manufacturing and Side Chain Modification

**Figure 1:**
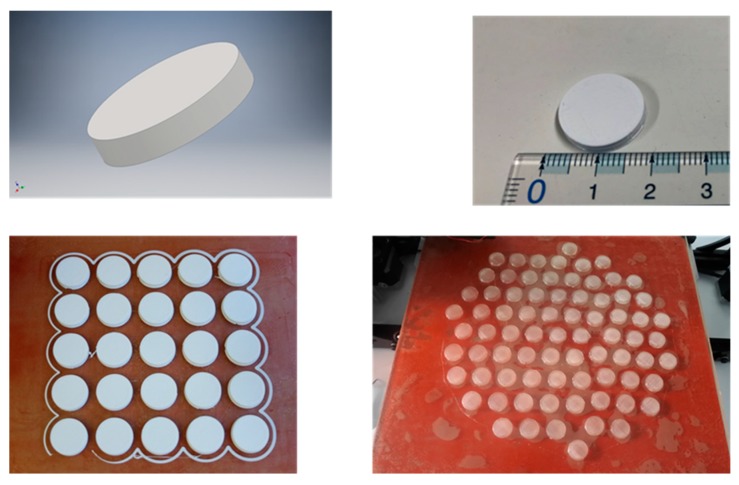
Top left STL (standard tessellation language) file format view of the polyvalent test plate (PVTP), top right printed PVTP showing size, bottom left 25 PVTP print batch on Lulzbot Mini, and bottom right 50 PVTP print batch on Lulzbot Taz 5.

In our experiments, PLA filaments were pre-formed with a localized heating cylinder so the filaments were melted. Then, the filament was 3D printed at 215 °C through a 0.4 mm stainless steel nozzle, using a layer height of 50 µm or 75 µm. The infill used was 100%, ensuring that porosity arises from the small spaces between the extruded filaments and any cavities between individual polymer molecules in the extruded filament. An infill of 100% also ensures high mechanical strength and that the reactant solution does not enter the printed PVTP and break down inter-layer cohesion. These 3D printed PLA polymer PVTPs of 14 mm diameter and 3 mm height provided the base for chemical modification, as shown in Figure 1.

**Figure 2:**
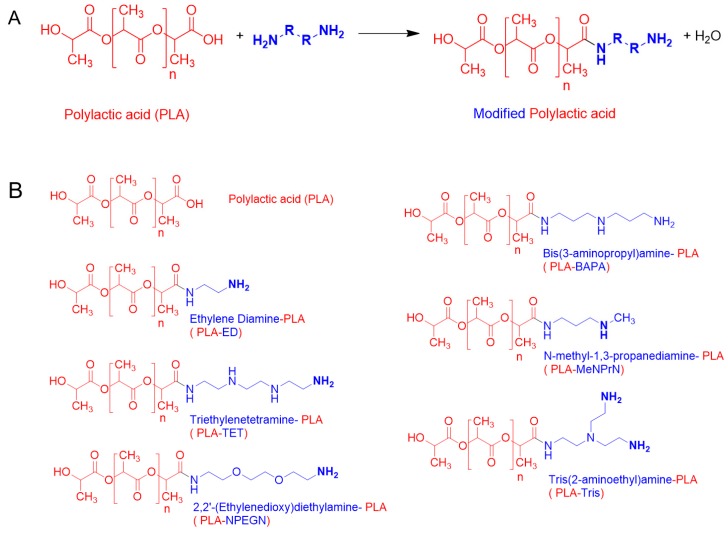
(**A**) Surface modification reaction for PLA with amines. (**B**) Chemical structure of PLA and the PLA-based amine-derivatives.

PLA PVTPs were chemically modified using different amines as side chains. This is unique to our study, because these 3D printed PLA PVTPs containing amine side chains have not been previously achieved. The concept of our modifications is based on the work of Haddad et al. [25]. The chemical reaction used in the surface modification is given in Figure 2A, and the structures of the modified PVTPs are presented in Figure 2B. PLA as a base polymer has advantageous mechanical properties and thermoplastic properties [36]. Due to these favorable attributes, it has been widely used in FDM based 3D printing processes.

The amines were chosen to present hydrophilic side chains of different natures: PLA-ED presents a short spacer with a primary amine head group, PLA-NprN a spacer and a secondary amine head group, PLA-TET and PLA-BAPA oligo-amino chains, PLA-NPEGN a short PEG spacer, and finally PLA-Tris a ramified di-amine head group.

Measurement with a digital micrometer before and after treatment showed an isotropic decrease in diameter and height of 50 µm, showing that 25 µm etching occurs at each surface.

### 3.2. Material Structure Characterization

#### 3.2.1. FT-IR Characterization of Chemical Modifications

**Figure 3:**
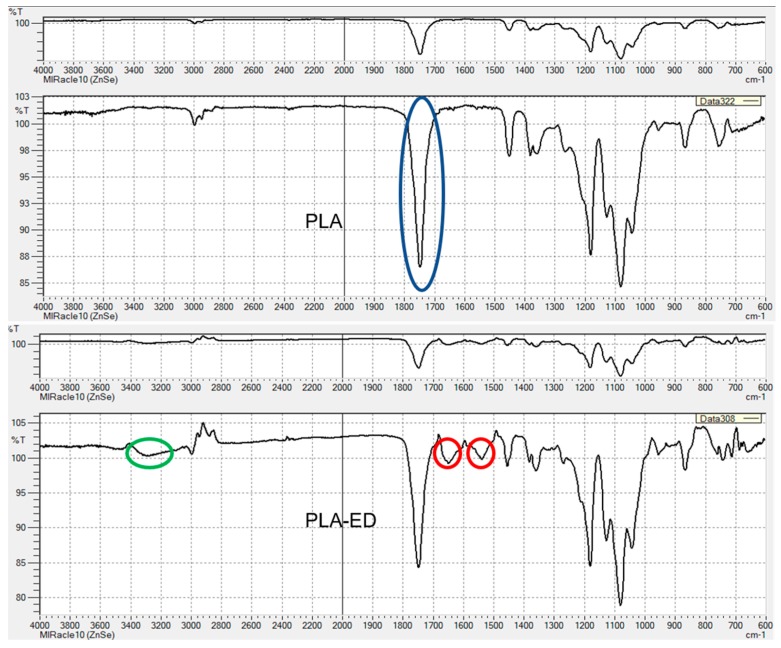
FT-IR spectra of PVTPs of PLA and PLA-ED. The ester C=O band is highlighted by a blue oval, the amide C=O bands by red circles, and the amide NH band by a green oval. From the presence of only the ester C=O stretch in PLA and the presence of both C=O amide and NH amide bands in the PLA-ED spectra, it is clear that the amide functionalization reaction has occurred, as was previously observed by time of flight secondary ion mass spectrometry (TOF-SIMS) for the surface modification of poly-ethylene-terephthalate (PET) by adenosine mono-phosphate (AMP) under the same conditions as in the current study [24].

**Figure A1:**
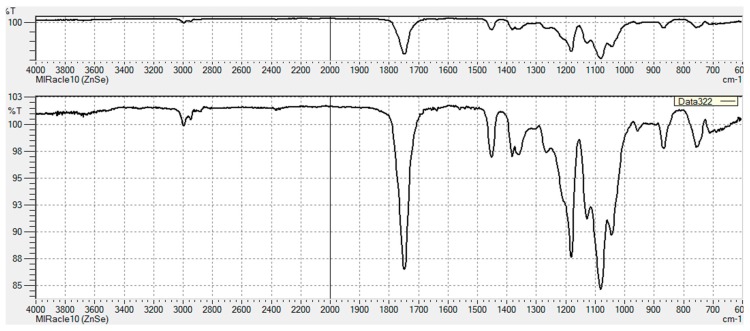
PLA.

**Figure A2:**
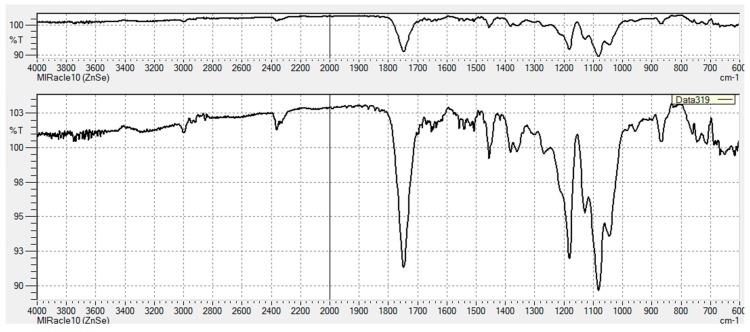
PLA-BAPA.

**Figure A3:**
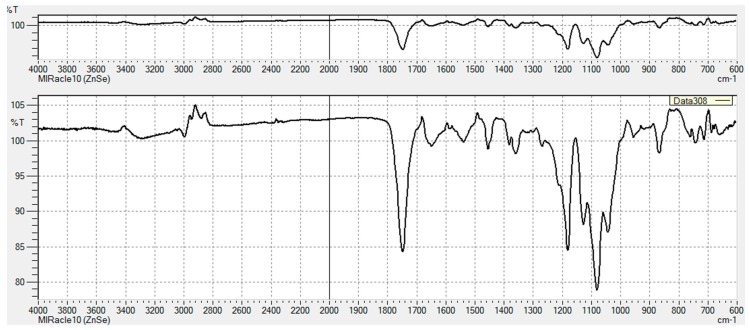
PLA-ED.

**Figure A4:**
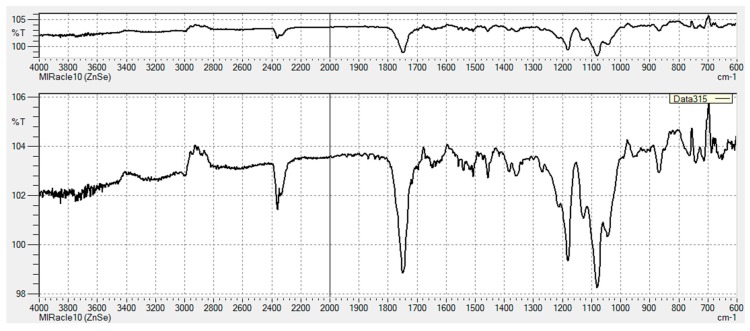
PLA-TET.

**Figure A5:**
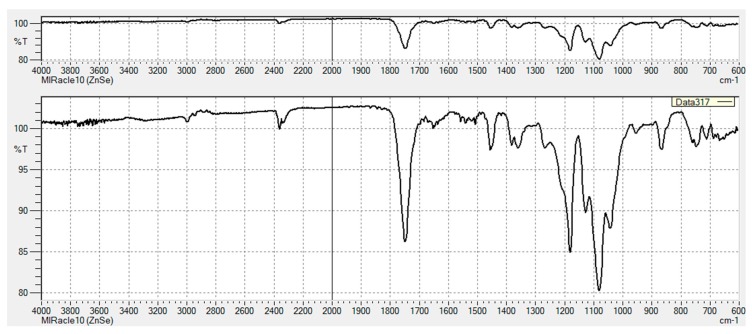
PLA-MeNprN.

**Figure A6:**
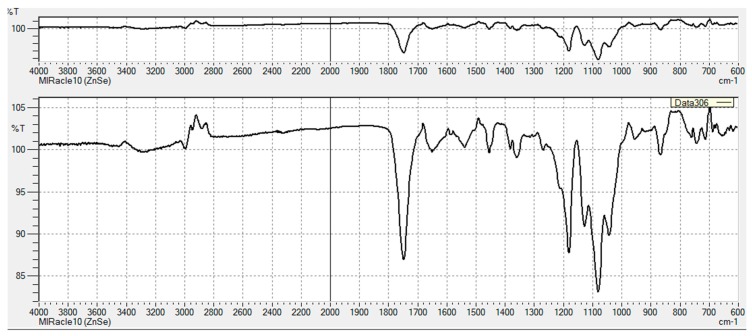
PLA-Tris.

**Figure A7:**
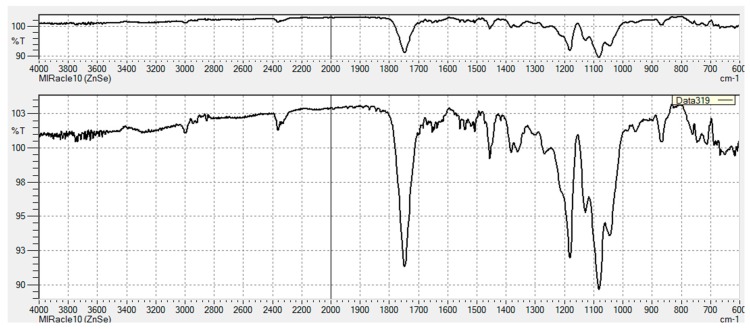
PLA-NPEGN.

The FT-IR spectra of PLA unmodified and PLA-ED are given in Figure 3, and FT-IR spectra for all modified PVTPs are given in Appendix A, Figure A1, Figure A2, Figure A3, Figure A4, Figure A5, Figure A6 and Figure A7. As expected, the spectrum of PLA is characterized by a weak C–H stretch band at 3000 cm^−1^ and a very strong C=O ester stretch at 1750 cm^−1^. For PLA-ED, additional C=O amide bands are observed at 1550 cm^−1^ and 1650 cm^−1^, and the presence of an amide stretch can only come from the formation of a covalent bond between the PLA polymer and the ethylene diamine. This is further confirmed by the presence of an N–H amide stretch band at 3300 cm^−1^. As expected from the fact that only surface modification is occurring, the intensities are much lower than that of the ester C=O band.

#### 3.2.2. Contact Angle Measurement

**Figure 4:**
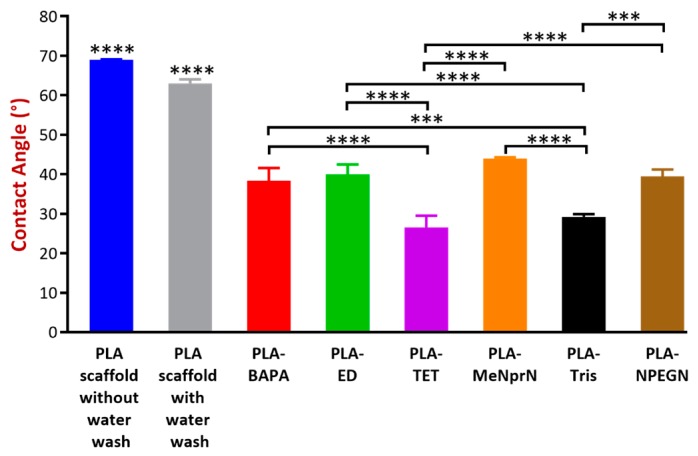
Contact angle values for the various PLA and chemically modified PVTPs. Data are expressed as means ± SD. Experiments were performed in triplicate, *n* = 3. Contact angle (°) values can be sorted in a decreasing order: PLA PVTP without water wash (69 ± 0.1°) = PLA PVTP with water wash (69 ± 0.1°) > PLA-MeNprN (45 ± 1.3°) > PLA-ED (41 ± 0.3°) > PLA-NPEGN (40 ± 1.1°) > PLA-BAPA (39 ± 0.5°) > PLA-Tris (30 ± 1.2°) > PLA-TET (26.5 ± 3.0°). The PLA PVTP with and without water wash have statistically significant differences in the contact angle values in comparison with the modified PLA based samples. PLA-TET and PLA-Tris modifications have statistically significantly lower results in comparison with the other modified PLA based samples. Error bars represent SD; *, **, and *** indicate statistically significant differences at *p* < 0.05, *p* < 0.01, and *p* < 0.001, respectively. In general, surfaces with contact angles of less than 90° are considered hemato-compatible.

The contact angle values obtained are given in Figure 4. The contact angle measurement results can be sorted based on a decreasing volume: PLA PVTP without water wash = PLA PVTP with water wash > PLA-MeNprN > PLA-ED > PLA-NPEGN > PLA-BAPA > PLA-Tris > PLA-TET. The contact angle values of our PVTPs were between 26.5 ± 3.0° and 69 ± 0.1°, showing that all the PVTPs are hydrophilic and that the chemical modification by introduction of polar oligo-amine functions results in increasing hydrophilicity. As expected, the unmodified PVTPs show the highest contact angles.

#### 3.2.3. Scanning Electron Microscopy

**Figure 5:**
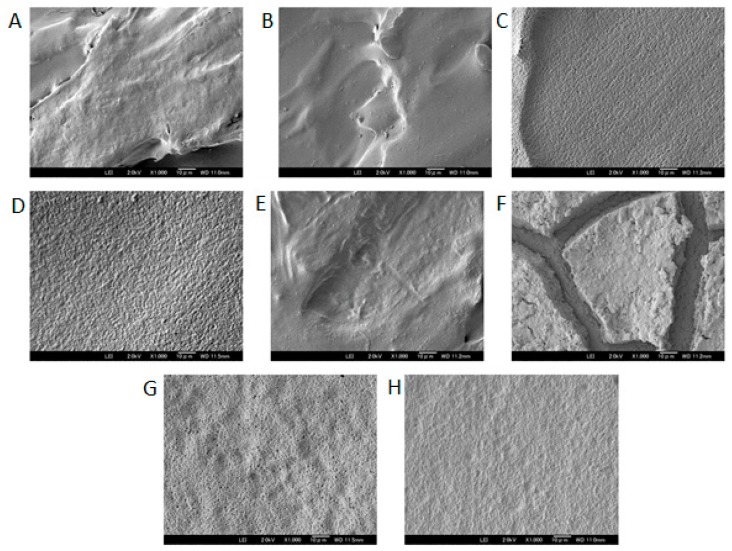
Surface morphology, and pore structure of the 3D printed PLA PVTPs were characterized using scanning electron microscopy (SEM). Magnification is ×1000, scale bar is 90 µm. (**A**) PLA with no treatment, (**B**) PLA with water wash, (**C**) PLA-BAPA, (**D**) PLA-ED, (**E**) PLA-TET, (**F**) PLA-MeNprN, (**G**) PLA-Tris, and (**H**) PLA-NPEGN.

The surface of the PLA PVTP and its modifications were tested using a scanning electron microscope. In our study, both the untreated surface and the deionized water washed surfaces had the general aspect of a stepped surface, with some small deviation from planarity for the untreated surface. After washing the surface appeared smoother, correlating with the change in the contact angle. For the six treated surfaces shown in Figure 5 below (C:PLA-BAPA, D:PLA-ED, G:PLA-Tris, and H:PLA-NPEGN), all appeared flattened with respect to the initial surface, however all showed a large number of indentations. PLA-Tris showed ca 1 µm pore diameters. In the case of the TET modified surface, the overall aspect was close to that of the untreated printed surface, and small indentations were visible with diameters of ca 100–200 nm. Finally, in the case of the MeNprN modified surface, etching appeared to have occurred and the surface was separated into iceberg-like systems overlying a flat surface. This confirms that the surface of the 3D printed PLA PVTPs had been modified, which confirms the formation of separated plates on the printed surface.

#### 3.2.4. Optical Microscopy Roughness Measurement

**Figure 6:**
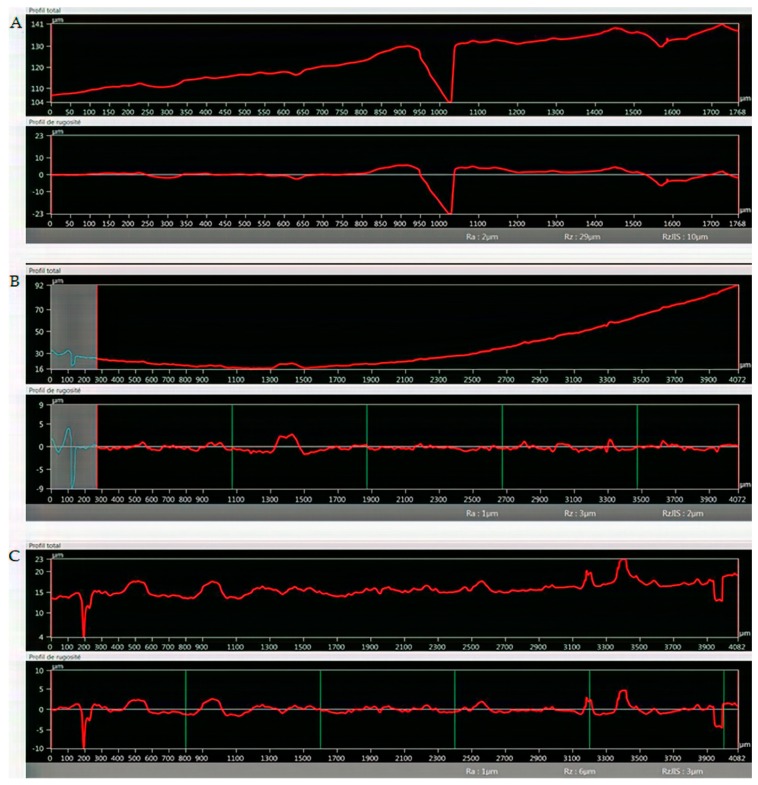
Line sections through printed PVTPs. (**A**) PLA unmodified 1768 µm length, (**B**) PLA-ED 4082 µm length, and (**C**) PLA-NPEGN 4082 µm. Rz values of 29 µm for PLA, 3 µm for PLA-ED, and 6 µm for PLA-NPEGN are observed.

**Figure A8:**
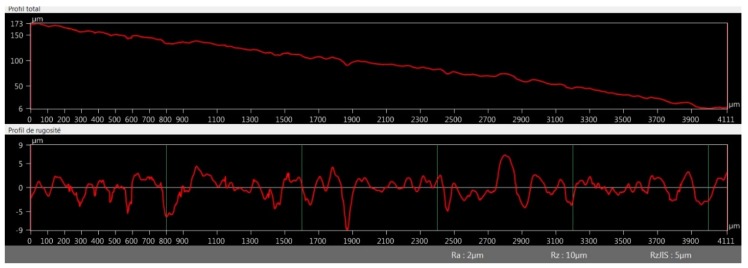
Line section PLA-MeNPrN.

**Figure A9:**
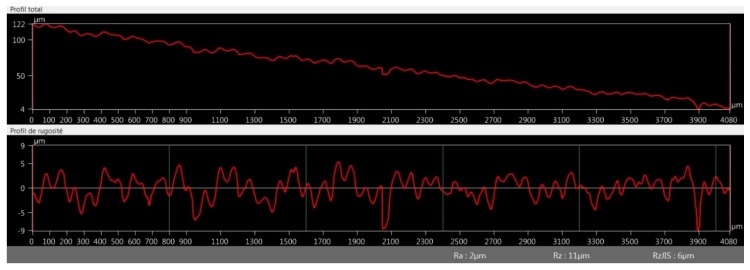
Line section PLA-BAPA.

**Figure A10:**
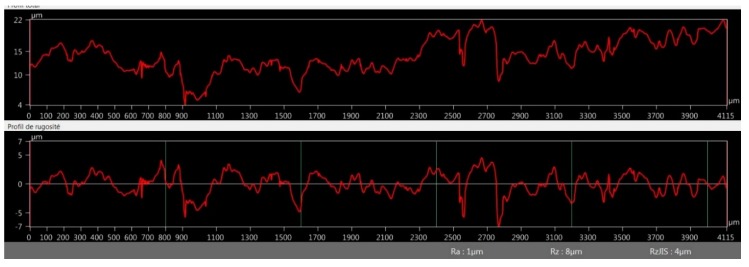
Line section PLA-Tris.

In Figure 6, line sections for PVTPs of unmodified PLA, PLA-Ed, and PLA-NPEGN are given, and other modifications of line sections can be seen in Appendix B, Figure A8, Figure A9 and Figure A10. These were obtained using a Keyence VHX 6000 optical microscope. Using the internal image processing program, Rz values of 29 µm for PLA, 3 µm for PLA-ED, and 6 µm for PLA-NPEGN were observed. The values of the PLA results from the presence of a void of about 20 µm between two extruded polymer filaments, and small depressions at 400 µm intervals arising from other filaments, were less than 5 µm in depth. The surfaces of modified PVTPs were generally quite flat with small pits and protrusions present in the modified samples, which was in agreement with the morphology observed using SEM. No systematically repeated depressions at 400 µm distances were observed. This was in agreement with the observation that 25 µm was etched from the PVTP during modification.

#### 3.2.5. Positron Annihilation Lifetime Spectrometry

**Figure 7:**
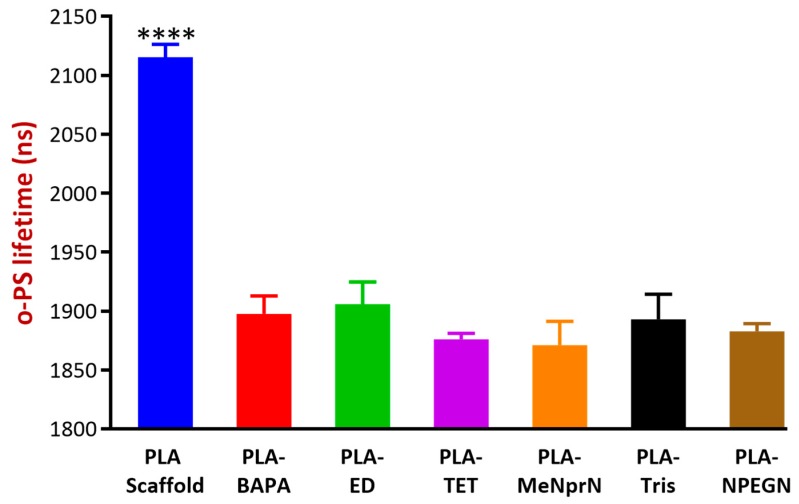
Average discrete positronium triplet state (o-Ps) lifetimes of various samples. A higher o-Ps lifetime represents higher free volume holes in the structure of the PLA based PVTPs. Values are presented as means ± SD. Experiments were performed in triplicate, *n* = 3. PLA PVTP has statistically significantly different o-PS lifetime values compared to the modified PLA based samples. In comparison, there is not a statistically significant different between the modified samples. BAPA = polylactic acid-bis(3-aminopropyl)amine; ED = polylactic acid-ethylenediamine; TET = polylactic acid-triethylenetetramine; MeNprN = polylactic acid-*N*-methyl-1,3-propanediamine; Tris = polylactic acid-tris(2-aminoethyl) amine; and NPEGN = polylactic acid- 2,2′-(Ethylenedioxy)diethylamine. Error bar represents SD; **** indicate statistically significant differences at *p* < 0.0001.

Figure 7 illustrates the average discrete o-Ps lifetime values of various samples. The higher lifetime values indicate higher free volume holes between the polymeric chains, and also residual space between filaments. The results showed that the modified polymeric bases (BAPA through NPEGN) were of similar o-Ps lifetimes, thus were free volume holes, while the PLA PVTP represented significantly higher o-Ps lifetimes. Based on our results, after the modification, PLA-ED and PLA-Tris contained the most free volume in their structures. PLA-MeNprN had the least free volume in its structure. The latter could be explained by the fact that through modification, the amines could be built into the polymeric chains, resulting in smaller free volumes between chains and especially between polymer molecules in the filaments.

### 3.3. Cytocompatibility Experiments

#### 3.3.1. MTT Cell Viability Test

A prolonged cell viability test was utilized in our work to gain information about the cytocompatibility of our PLA PVTPs. The samples were incubated in the cell culture medium for four, eight, and 12 days, and the Caco-2 cells were treated by this medium [37,38]. This method differs from the original MTT assay because the inhibitory concentration (IC_50_) was not measured, but the cell viability was calculated in comparison with a negative (DMME medium). In the works of Kinnari and Chessa et al., Caco-2 cell lines were the gold standard for the cytotoxicity examinations of orthopedic and breast implants [39,40]. Caco-2 cell lines are a model of the intestinal barrier, because numerous attributes are the same as in the mature enterocyte.

**Figure 8:**
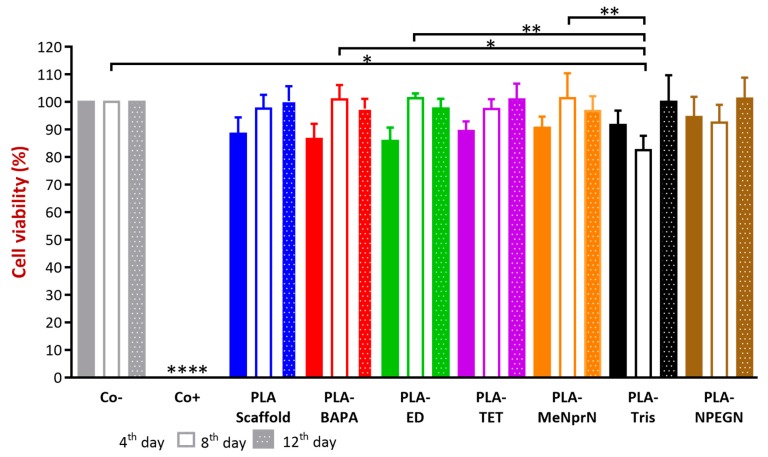
Prolonged cytotoxicity effects of the PLA based chemically modified PVTPs on CaCo-2 cells determined by an MTT cell viability test on the 4th, 8th, and 12th days. Cell viability was expressed as the percentage of untreated control in the case of PLA-based chemically modified PVTPs. The positive control was Triton X 100 (10% *w/v*), which has significantly different cell viability results compared to the Co− and the examined samples. Data are means of three independent experiments ± S.D. We compared cytotoxic values and found none of the samples decreased significantly in cell viability compared to the untreated control. Error bars represent SD; *, **, and **** indicate statistically significant differences at *p* < 0.05, *p* < 0.01, and *p* < 0.001, respectively.

The results are expressed as the percentage of negative or untreated control (Co−). As a positive control (Co+) Triton-X 100 (10% *w/v*) solubilizing agent was used, which resulted in significant differences from the other examined samples. We compared cytotoxic values and found none of the samples decreased significantly in cell viability compared to the untreated control (Figure 8.) Based on our MTT assay, PLA-TET cytotoxicity did not decrease under 100% in comparison with the negative control. PLA-ED, PLA-BAPA, PLA-Tris, and PLA-NPEGN cell viability values were higher than 90% in the case of prolonged exposure. Based on the ISO 10993-5:2009(E) standard, if the relative cell viability is higher than 70% in comparison with the control group (100%), the materials can be considered non-cytotoxic [41]. According to this regulation, all our PLA PVTPs qualified as cytocompatible.

#### 3.3.2. Biofilm Formation

**Figure 9:**
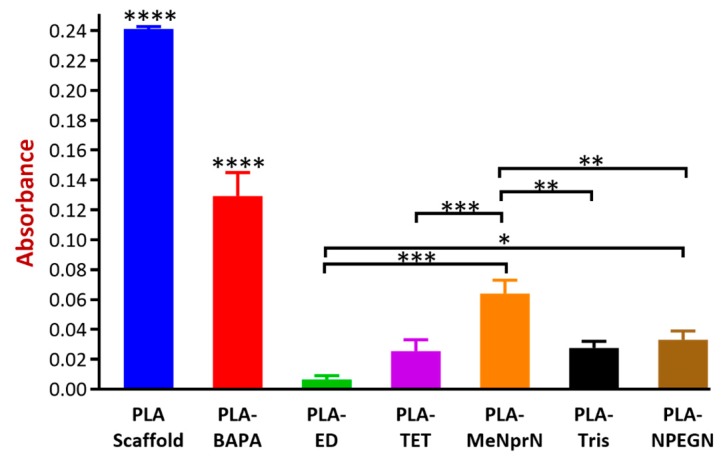
Biofilm formation results are presented as an absorbance value plotted against the examined samples, and all absorbance results were under 0.24. Values are presented as means ± S.D. Experiments were performed in triplicate, *n* = 3. PLA PVTP and BAPA modification have significantly higher absorbance results than other modifications. MeNprN has higher absorbance results than 0.05 but TET, Tris, NPEGN, and ED have lower absorbance results than 0.05. In comparison with the other PVTPs, ED resulted in the smallest absorbance value. Error bars represent SD; *, **, ***, and **** indicate statistically significant differences at *p* < 0.05, *p* < 0.01, *p* < 0.001, and *p* < 0.0001, respectively.

Our experiments were based on the fact that the formed biofilm can be visualized with a crystal violet (CV) stain, and the absorbance of the dye can be measured. The measured absorbance values were in correlation with the amount of biofilm in the implants [34,42]. All of the results of biofilm formation were under a 0.24 absorbance value. PLA PVTP and BAPA modification result in significantly higher absorbance results than other modifications. MeNprN has higher absorbance results than 0.05, but TET, Tris, NPEGN, and ED have lower absorbance results than 0.05. In comparison with the other PVTPs, ED resulted in the smallest absorbance value. (Figure 9). According to the classification from Marcos-Zambrano et al., low biofilm forming ability occurs with absorbance values under 0.44, moderate biofilm forming ability occurs between absorbance values of 0.44–1.17, and high absorbance forming ability occurs with absorbance values above 1.17. Based on this classification, all of our implants showed low biofilm forming ability [43]. There are some significant differences among the chemically modified PLA PVTPs because PLA-ED resulted in the lowest biofilm formation, however PLA-BAPA and PLA PVTP represented the highest biofilm formation.

## 4. Discussion

Nowadays, 3D printing technologies for medical purposes are mainly focusing on bioprinting, dental applications, orthopedic applications, and development of modified release oral drug delivery systems [2]. In our study, morphological characterization and in-vitro anti-fungal and cytocompatibility tests of chemically modified PLA-based 3D printed PVTPs are presented. In our research, surface modification of PLA polymer using oligoamines introduced better anti-infective properties to the polymer without altering its other properties, however in the paper by Tappa et al., addition of anti-infective agents was associated with a decrease in the mechanical properties of PLA, even though it retained its anti-infective properties [44].

Polyvalent test plates were designed for investigation of the physical, chemical, and in-vitro biological properties of FDM printed materials, thus the term polyvalent in this article refers to the use of a single structure for testing of a wide range of properties. The advantage of such a PVTP was the exclusion of effects due to differing geometries, and enabling of the direct comparison between test results. A polyvalent can be used in many situations—chemistry, physics, mechanics, biology, and cell growth, and even biofilm formation.

The reason the printed PVTPs were chemically modified was to increase their favorable surface properties and biocompatibilities. These chemical modifications were verified by FT-IR spectroscopy, showing introduction of amide and amine functions. The FT-IR data was upgraded, highlighting the bands corresponding to the ester C=O, amide C=O, and N-H stretches. The intensities of the amide C=O and N–H are relatively weak as they are surface bonds. Other methods are able to show amide formation, but none are applicable to surface modification. For example, 13C NMR would not detect C at less than 0.1% [45]. Other methods would not show the formation of covalent bonds.

There is clear evidence for the influence of surface topographical and wetting characteristics on macromolecular and cellular levels at implant interfaces [46]. The polymer ester backbone was surface modified by a series of ramified and linear oligoamines to increase the hydrophilicity of the PVTP surfaces and introduce positive charge. These hydrophilic surface modifications were certified by contact angle measurement, which is crucial based on work by Song et al. because wettability of PVTPs may be affected by chemical modification [47]. However, as ethylene diamine is a small and highly water soluble molecule, it would be expected to be washed off the surface. This is not the case, as the contact angle remains constant after washing vigorously. The surface wettability of biomaterials determines the biological events at the biomaterial/host interface [46]. Wettability is modulated by surface chemistry, as was shown in our work and in the articles of Nasrin et al. and Mi et al., because a large range of solid characterization methods can be used for the qualification of different 3D printed polymers in these articles [28,48]. There is also evidence regarding the pH dependence of the contact angle, but the method is as yet not fully proved [49].

The roughness of our PLA based PVTPs was determined by scanning electron microscopy. However, the result of surface scanning depends on many parameters (i.e., the applied filters, the evaluation areas, etc.). The assessment of micron, submicron, and nano-roughness of different implant surfaces is inevitable. Nevertheless, there are only a few standardized methods for the correct determination of implant surfaces [46], although the prediction of the biological performance of different implants in the human body is essential.

Positron annihilation spectroscopy (PALS) measures the supramolecular structure of polymeric-based implants, which can give correct information about the pore sizes and the porosity of the surfaces. Approximately the upper 100 μm of the surface can be examined with this method, and deviations can be seen in a nano-dimensional range. This method is based on one of Einstein’s principles, and the detected o-Ps lifetime values are in correlation with the free volume holes of the samples, which enables the comparison of the loaded and unloaded delivery bases. The latter could be useful in the characterization of amorphous solid dispersions or solutions [50].

Comparing the results of PALS and SEM experiments, PLA-ED, PLA-TET, and PLA-Tris all showed high free volumes and the presence of numerous pits. For PLA-ED and PLA-Tris these were ca 1 µm in diameter, and for PLA-TET these pits were ca 100–200 nm in diameter. However, for MeNprN, few pits were present and the free volume was much reduced. Thus, there appeared to be a direct relationship between the free volume measured by PALS and the presence of large numbers of pits observed by SEM.

PLA showed good biocompatibility results and was biodegradable through hydrolytic degradation [47]. Our samples were stored in DMEM medium at 37 °C until the end of the 12th day, and these extracts were measured on days four, eight, and 12 on the Caco-2 cells. The incubation period was 30 min. The cytotoxicity test was harmonized with the ISO 10993-5 standard, but the incubation period was shorter [41]. There is clear evidence that PLA degrades into lactic acid and glycolic acid during storage, and different factors (pH, molecular weight, etc.) can influence this degradation [51]. Many studies have dealt with the degradation of PLA polymers, so the main focus of our study was not the investigation of PLA degradation but the cytotoxic and biofilm formation effects of the modified PLA samples stored in DMEM medium, where the samples may degrade. In-vitro cytotoxicity methods can be the first filter in the assessment of cytocompatibility, because the assay is sensitive, efficiently adaptable, and well reproducible [52]. MTT assay is a highly efficient screening test because only the viable cells are able to convert the dye through their mitochondrial enzymes. Van Tonder et al. revealed that the MTT dye alteration to formazan salt depends on the cells’ metabolic rate and number of mitochondria [19]. In spite of this limitation of MTT assay, it is still the most reliable and quickest test for the assessment of cytocompatibility [53]. Ramot et al. revealed that PLA can provoke an inflammatory reaction through implantation [54]. Silva et al., however, certified our result because they pointed out that these inflammatory responses, as adverse reactions caused by PLA samples, are extremely rare [55]. Based on the results of the MTT test our PLA samples proved to be cytocompatible.

Biofilm formation is also a compulsory element of cytocompatibility examination, based on the paper by Yang et al. In this research, 3D printed polylactide-*co*-glycolide and hydroxyapatite PVTPs were examined [56]. Biofilm formation can be associated with long-term implantation, which can lead to infections and antimicrobial resistance [34]. During implantation any kind of infection could be dangerous, because it may result in serious inflammation and the rejection of implants. However, biofilm formation can alter the structure of PLA and may cause liberation of different APIs in the case of 3D printed medicine, for example [54]. In our study, biofilm formation was performed with *Candida albicans* SC5314 reference isolate, because one of the most common biofilm forming fungi are *Candida* spp. [34] and other species of *Candida* can cause serious invasive candidiasis, with a mortality rate of about 45% [57]. Ideally, the measured absorbance is zero, which means that no kind of biofilm is formed on the implant by the reference isolate. According to the evaluation of biofilm formation, the chemically modified PLA-PVTPs are biocompatible.

In our paper, we demonstrated the importance of surface characterization and cytocompatibility investigations of 3D printed PLA- PVTPs and chemically modified PLA-based PVTPs. The amines as side chains can favorably alter the surface properties, wettability, and biofilm formation of PLA PVTP. Low biofilm formation ability and favorable cytocompatibility profiles were also presented in the case of prolonged exposure. There are connections between the type and the other properties of PLA PVTPs. Overall, the properties of 3D printing materials can be dramatically enhanced by the modification of base polymers. These implants may ensure a high possibility for the incorporation of different antimicrobial APIs. According to these results, PLA-ED, PLA-Tris, and PLA-TET PVTPs can show favorable surface and anti-infective properties. Therefore, these samples were selected for further in-vivo and/or human studies.

## 5. Conclusions

To conclude, we have successfully printed 3D PVTPs in runs of up to 50 units, and these have then been chemically functionalized at the surface by various amine head groups. The surface characterization confirms the modification. The various types of PVTP show no toxicity. Finally, we note that a suitable choice of the head group allows *Candida albicans* biofilm formation to be reduced by a factor of ten. Based on these in-vitro tests (MTT and biofilm formation tests), it can be concluded that more than one assay should be used to determine cytotoxicity, so as not to over or underestimate the cytocompatibility of 3D printed PVTPs. However, cytotoxicity data alone are not necessarily predictive of in-vivo issues, but alongside other experiments (contact angle, PALS, and SEM results) the in-vivo compatibility data may be estimated. This work is being extended to other functionalizations, including anionic head groups and other polymers such as PET or PMMA.

## Figures and Tables

**Table 1:** Printing characteristics for the plates and plate arrays used in the current work.

**Printer**	**Lulzbot Mini**	**Lulzbot Taz 5**
**Filament Type**	PLA	PLA
**Source**	Maker Shop France	Maker Shop France
**Filament Diameter; mm**	2.85	2.85
**Extruder Nozzle Diameter; µm**	350	350
**Infill Percentage**	100	100
**Extrusion Temperature (°C)**	215	215
**Bed Temperature (°C)**	60	60
**Layer Thickness µm**	50	75
**Print Speed mm/s**	50	50

## Appendix A

FT-IR spectra of polyvalent test platforms (PVTP).

## Appendix B

Line sections through printed PVTPs.
